# Rice CONSTITUTIVE TRIPLE-RESPONSE2 is involved in the ethylene-receptor signalling and regulation of various aspects of rice growth and development

**DOI:** 10.1093/jxb/ert272

**Published:** 2013-09-04

**Authors:** Qin Wang, Wei Zhang, Zhongming Yin, Chi-Kuang Wen

**Affiliations:** National Key Laboratory of Plant Molecular Genetics and National Center for Plant Gene Research (Shanghai), Institute of Plant Physiology and Ecology, Shanghai Institutes for Biological Sciences, Chinese Academy of Sciences, 300 Fenglin Road, Shanghai 200032, PR China

## Abstract

In *Arabidopsis*, the ethylene-receptor signal output occurs at the endoplasmic reticulum and is mediated by the Raf-like protein CONSTITUTIVE TRIPLE RESPONSE1 (CTR1) but is prevented by overexpression of the CTR1 N terminus. A phylogenic analysis suggested that rice OsCTR2 is closely related to CTR1, and ectopic expression of *CTR1p:OsCTR2* complemented *Arabidopsis ctr1-1*. *Arabidopsis* ethylene receptors ETHYLENE RESPONSE1 and ETHYLENE RESPONSE SENSOR1 physically interacted with OsCTR2 on yeast two-hybrid assay, and green fluorescence protein-tagged OsCTR2 was localized at the endoplasmic reticulum. The *osctr2* loss-of-function mutation and expression of the *35S:OsCTR2* ^*1–513*^ transgene that encodes the OsCTR2 N terminus (residues 1–513) revealed several and many aspects, respectively, of ethylene-induced growth alteration in rice. Because the *osctr2* allele did not produce all aspects of ethylene-induced growth alteration, the ethylene-receptor signal output might be mediated in part by OsCTR2 and by other components in rice. Yield-related agronomic traits, including flowering time and effective tiller number, were altered in *osctr2* and *35S:OsCTR2* ^*1–513*^ transgenic lines. Applying prolonged ethylene treatment to evaluate ethylene effects on rice without compromising rice growth is technically challenging. Our understanding of roles of ethylene in various aspects of growth and development in *japonica* rice varieties could be advanced with the use of the *osctr2* and *35S:OsCTR2* ^*1–513*^ transgenic lines.

## Introduction

Ethylene, a gaseous plant hormone, regulates many aspects of plant growth and development, such as responses to stress and pathogens, fruit ripening, senescence, (Anderson *et al.*, 2004), and cell elongation (Alonso and Granell, 1995; Hua and Meyerowitz, 1998; Alexander and Grierson, 2002). Many studies have used mainly dicotyledonous plants to investigate the effects of ethylene. In contrast, knowledge of the roles of ethylene in lower plants and monocotyledonous plants is relatively scarce.

Previous studies of the ethylene effects on rice have focused mostly on flooding responses in a few specific varieties of the *inidca* rice cultivar. In varieties of *indica* floating rice (also called deep-water rice), flooding induces a burst of ethylene biosynthesis, which promotes gibberellin biosynthesis and abscisic acid degradation (van der Knaap *et al*., 2000; Saika *et al.*, 2007; Fukao and Bailey-Serres, 2008*a*). As a result, internodal elongation is facilitated and the rice plant is able to grow above the water and survive. Two quantitative trait loci responsible for ethylene-induced internodal elongation in the deep-water rice varieties have been identified; they are members of the *ETHYLENE RESPONSE FACTOR* (*ERF*) family, namely *SNORKEL1* (*SK1*) and *SK2*. Gibberellin treatment can replace ethylene treatment to induce internodal elongation in deep-water rice varieties, so rapid rice growth with flooding can be independent of ethylene (Hattori *et al.*, 2009).

Unlike deep-water rice, the *indica* variety FR13A does not produce an elongated shoot and can survive with complete submergence in water. FR13A carries the *SUBMERGENCE1A* (*SUB1A*) gene that encodes an ERF protein conferring submergence tolerance (Xu *et al.*, 2006; Jung *et al.*, 2010). *SUB1A* expression is inducible by ethylene on submergence, and the DELLA proteins SLENDER RICE1 (SLR1) and SLENDER RICE LIKE1 (SLRL1) accumulate to inhibit shoot elongation (Fukao and Bailey-Serres, 2008*b*). The growth inhibition reduces energy consumption to facilitate growth recovery after submergence (Fukao *et al.*, 2006). The *SUB1A* locus is absent in *japonica* varieties and is not functional in many *indica* varieties that are intolerant of submergence (Xu *et al.*, 2006). Although the roles of ethylene in flooding responses are clearly revealed in deep-water rice and submergence-tolerant *indica* varieties, regular rice varieties do not show the rapid shoot elongation or growth inhibition on flooding. The general roles of ethylene in many aspects of rice growth and development remain to be fully addressed.

*Arabidopsis* ETHYLENE INSENSITIVE2 (EIN2) and EIN3 are components in the ethylene signal transduction pathway promoting ethylene responses. Transformation studies show opposite effects of rice *OsEIN2* antisense expression and *EIN3-LIKE1* (*OsEIL1*) overexpression on seedling height and primary root growth (Jun *et al.*, 2004; Mao *et al.*, 2006). Overexpressing the rice ethylene receptor-like gene *ETHYLENE RESPONSE2* (*OsETR2*) moderately alleviated seedling elongation and root growth alterations induced by 1-aminocyclopropane-1-carboxylic acid (ACC) (Wuriyanghan *et al.*, 2009). *Arabidopsis* REVERSION- TO-ETHYLENE SENSITIVITY1 (RTE1) is a Golgi/endoplasmic reticulum (ER) protein that promotes the signal output from the ETHYLENE RESPONSE1 (ETR1) ethylene receptor. Treatment with the ethylene blocker 1- methylcyclopropene (1-MCP) and overexpression of rice *REVERSION-TO-ETHYLENE SENSITIVITY1 HOMOLOG1* (*OsRTH1*) each effectively prevented many aspects of ethylene-induced alterations in growth and gene expression (Zhang *et al.*, 2012). These studies suggest that the ethylene signalling machinery is conserved in *Arabidopsis* and rice.

CONSTITUTIVE TRIPLE-RESPONSE1 (CTR1) is a key component in *Arabidopsis* mediating the ethylene-receptor signal output, and *ctr1* loss-of-function mutations result in a constitutive ethylene response (Huang *et al.*, 2003). Isolation of rice mutants exhibiting a constitutive ethylene response will advance our knowledge of the effects of ethylene on rice throughout development. Here, we showed by a cross-species complementation test, mutant phenotype analyses, and dominant-negative effects with the expression of *35S:OsCTR2* ^*1–513*^ that encodes the OsCTR2 N terminus that rice OsCTR2 is closely related to CTR1 and negatively regulates ethylene signalling. The *osctr2* allele did not promote all aspects of ethylene-induced growth alterations, so OsCTR2 was not the only component mediating the ethylene-receptor signal output. Ethylene effects on aspects of rice growth and development could be evaluated with the use of the *osctr2* and *35S:OsCTR2* ^*1–513*^ transgenic lines.

## Materials and methods

### Plant materials and growth conditions

The wild-type *japonica* rice cultivars used were ZH11 and Dongjing (DJ), and the *osctr2* allele was in the DJ background. The *osctr2* mutant was from Dr Gyheung An (Kyung Hee University, Korea) (Jeon *et al.*, 2000; Jeong *et al.*, 2006) and was confirmed by PCR genotyping. Conditions for rice seed germination and growth were as described previously (Zhang *et al.*, 2012). *Arabidopsis* seeds were stratified for 72h before germination; seedling phenotypes were scored after 80h of germination at 22 °C in the dark or 7 d of germination with illumination (16h light/8h dark). For gas treatment, *Arabidopsis* or rice seedlings were grown in an air-tight container with ethylene (100 µl l^–1^) or 1-MCP (5 µl l^–1^). 1-MCP was prepared according to the manufacturer’s instructions (Rohm & Haas China, Beijing), and the concentration was determined by gas chromatography with a flame ionization detector (Zhang *et al.*, 2012). *Arabidopsis* seedlings were treated for 80h (etiolated seedlings) or 7 d (light-grown seedlings), and etiolated rice seedlings for 7 d after germination or 4 d for light-grown seedlings (Xie *et al.*, 2006; Zhou *et al.*, 2007; Zhang *et al.*, 2012). For the senescence test, rice leaf segments were treated for 4 d (Zhang and Wen, 2010). Rice plants were cultivated in an experimental station in Shanghai. Ethylene concentration was determined by gas chromatography/flame ionization as described previously (Zhang *et al.*, 2012).

### Phylogenetic analysis

Plant CTR1-related proteins were searched for using BLAST (http://blast.ncbi.nlm.nih.gov/Blast.cgi) with *Arabidopsis* CTR1 as the query sequence. Redundant and short sequences were excluded. The sequences were aligned using ClustalX version 2.1 (Larkin *et al.*, 2007), and a neighbour-joining tree was generated using MEGA5.0, with a bootstrap setting of 1000 (Tamura *et al.*, 2011).

### Transgenes and clones

To clone the *Arabidopsis CTR1* promoter, the primer set AtCTR1OF and AtCTR1PR was used for PCR cloning. All primer sequences used for cloning are available in Supplementary data S1 at *JXB* online. Rice *OsCTR2* and *OsCTR3* cDNA clones were from the Rice Genome Research Center, National Institute of Agrobiological Sciences, Japan. The primer set OsCTR2-F and OsCTR2-R was used to generate the *OsCTR2* cDNA fragment for cloning *CTR1p:OsCTR2*. *CTR1p:OsCTR2* was transformed into Arabidopsis *ctr1-1* for a cross-species complementation test. The primer set OsCTR2-N-F and OsCTR2-NR was used to PCR clone the *OsCTR2* ^*1–513*^ fragment, with rice genomic DNA used as a template. The ER marker ER-rk has been described previously (Nelson *et al.*, 2007; Zhang *et al.*, 2012). *35S:OsCTR2* ^*1–513*^ transgenic rice lines were obtained by *Agrobacterium*-mediated transformation into rice callus (ZH11 variety), and the resulting transgenic lines were obtained from independent calli. Phenotyping for *35S:OsCTR2* ^*1–513*^ transgenic lines was performed in T3 or higher generations.

### Quantitative reverse transcription-PCR (qRT-PCR) analysis

qRT-PCR analysis of gene expression involved the use of a StepOne Real-Time PCR System (ABI) with a SYBR Premix Ex *Taq* real-time RT-PCR kit (Takara). The primer set for *ETHYLENE RESPONSE FACTOR1* (*ERF1*) expression has been described previously (Liu and Wen, 2012*b*). The primer sets for qRT-PCR are available in Supplementary data S1. Expression of actin (rice) and ubiquitin (*Arabidopsis*) were used for internal calibration. To measure the transcript copy number for *CTR1* and *OsCTR2* in *Arabidopsis* expressing *CTR1p:OsCTR2*, cDNAs for *CTR1* and *OsCTR2* were used as templates, serially diluted, and a standard curve for the cDNA copy number was drawn (*R* ^2^ ≥ 0.99). Total RNA was reversed transcribed with the use of oligo(dT), and *CTR1* and *OsCTR2* copy numbers were estimated against the standard curve by qRT-PCR.

### Laser-scanning confocal microscopy

Laser-scanning confocal microscopy for subcellular localization of fluorescently labelled proteins involved use of an Olympus FluoView FV1000 and FV10-ASW1.7 Viewer for data acquisition at the Core Facility Center of the Institute of Plant Physiology and Ecology, Shanghai Institutes for Biological Sciences. Transgenes that expressed the fusion proteins were delivered by particle bombardment to onion epidermal cells or by *Agrobacterium* infiltration to tobacco leaf epidermis.

### Statistical analyses

For *Arabidopsis* seedling hypocotyl measurement, at least 30 individual seedlings were measured and the hypocotyl length was described as mean ±SD. Gene expression analysis with qRT-PCR involved three independent biological samples, with each measurement repeated three times, and data are described as means ±SEM. At least 28 rice seedlings were scored for measurement of seedling height, primary root length, number of adventitious roots, and coleoptile length, and data are described as means ±SD. Chlorophyll *a* content was determined from six independent measurements, with leaf segments from at least three independent rice seedlings pooled for each measurement. The sample size for agronomic traits of rice plants is shown in Fig. 6. Student’s *t*-test was used for comparing paired means and Scheffe’s test for multiple means (α = 0.01).

### Yeast two-hybrid assay

A yeast two-hybrid assay and β-galactosidase activity measurement were performed as described previously (Clark *et al.*, 1998; Xie *et al.*, 2006). cDNA fragments encoding ETR1^331–729^ and ETHYLENE RESPONSE SENSOR1 (ERS1)^130–613^ were each cloned in pBTM116 and co-expressed with OsCTR2^1–513^ (cloned in pGADT7) or CTR1^53–568^ (in pGAD424) (Clark *et al.*, 1998) in the yeast strain L40. β-Galactosidase activity was determined by OD_578_ (the OD was read every second for 600 s) from 8–10 independent yeast transformation lines, with chlorophenol red–β-d-galactopyranoside as the substrate, and the data are shown as means ±SD. For 5-bromo-4-chloro-indolyl-β-d-galactopyranoside (X-gal) staining, yeast cells were grown on non-selective medium supplemented with X-gal (80mg l^–1^) for 24h at 30 °C.

## Results

### Phylogenetic analysis of CTR1-related proteins

To identify proteins related to *Arabidopsis* CTR1 in rice, we performed a BLAST search and identified 64 CTR1-related proteins from 25 plant species for phylogenetic analysis.

**Fig. 1.**
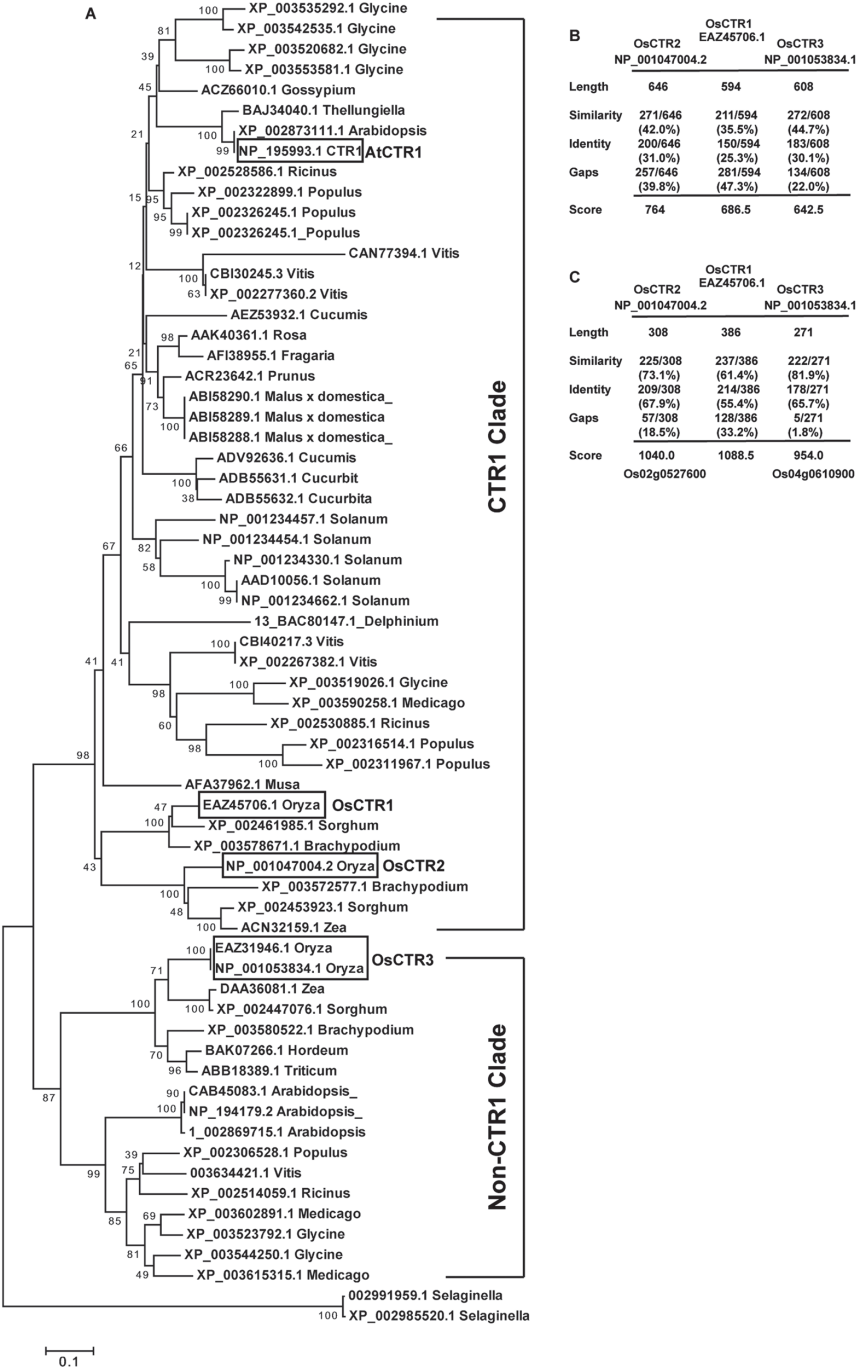
Sequence and phylogenetic analyses of CTR1-related proteins. (A) Phylogenetic tree of plant CTR1-related proteins with accession number and genus names. *Arabidopsis* CTR1 (AtCTR1) and rice OsCTRs (OsCTR1, OsCTR2, and OsCTR3) are boxed. (B, C) Protein sequence similarity and identity at the N terminus (B) and C terminus (C) of rice OsCTRs compared with that of *Arabidopsis* CTR1.

CTR1-related proteins of all plant species except *Selaginella* were classified into two clades (Fig. 1A). Within the CTR1 clade, *Arabidopsis* CTR1 and CTR1-related proteins from dicotyledonous plant species were in the same subclade, and OsCTR1/OsCTR2 and CTR1-related proteins from monocotyledonous plants were in another subclade. OsCTR3 was in the non-CTR1 clade, and, consistently, proteins from dicots and monocots were classified into two subclades. The phylogenetic analysis suggested that both OsCTR1 and OsCTR2 are more closely related to CTR1 than OsCTR3. *Selaginella* CTR1-related proteins appeared ancestral to CTR1-related proteins of seed plants; whether they acquired the ability to mediate ethylene-receptor signalling remains to be determined.

The *Arabidopsis* CTR1 N terminus interacts physically with the ethylene receptors ETR1 and ERS1 to mediate receptor signalling, and an excess amount of the CTR1 N terminus CTR1^7–560^, prevents receptor signalling and results in constitutive ethylene responsiveness. The CTR1 C terminus has a Ser/Thr kinase domain, and the kinase activity is essential for CTR1 functions (Clark *et al.*, 1998; Huang *et al.*, 2003; Qiu *et al.*, 2012). Analysed with the EMBOSS Needle Tool (http://www.ebi.ac.uk/Tools/psa/emboss_needle/), *Arabidopsis* CTR1 was found to share a higher degree of sequence similarity and identity at the N and C termini with OsCTR1 and OsCTR2 than with OsCTR3 (Fig. 1B, C).

### Expression of CTR1p:OsCTR2 complements Arabidopsis ctr1-1 mutation

To evaluate whether *OsCTR1* and *OsCTR2* have any roles in ethylene-receptor signalling, we ectopically expressed each gene in a *ctr1-1* loss-of-function mutant for complementation testing. The OsCTR1 sequence was annotated without mRNA evidence. With the annotation information, efforts to clone the *OsCTR1* cDNA by RT-PCR were invalid, and the presence of the *OsCTR1* locus remains to be experimentally determined.

**Fig. 2.**
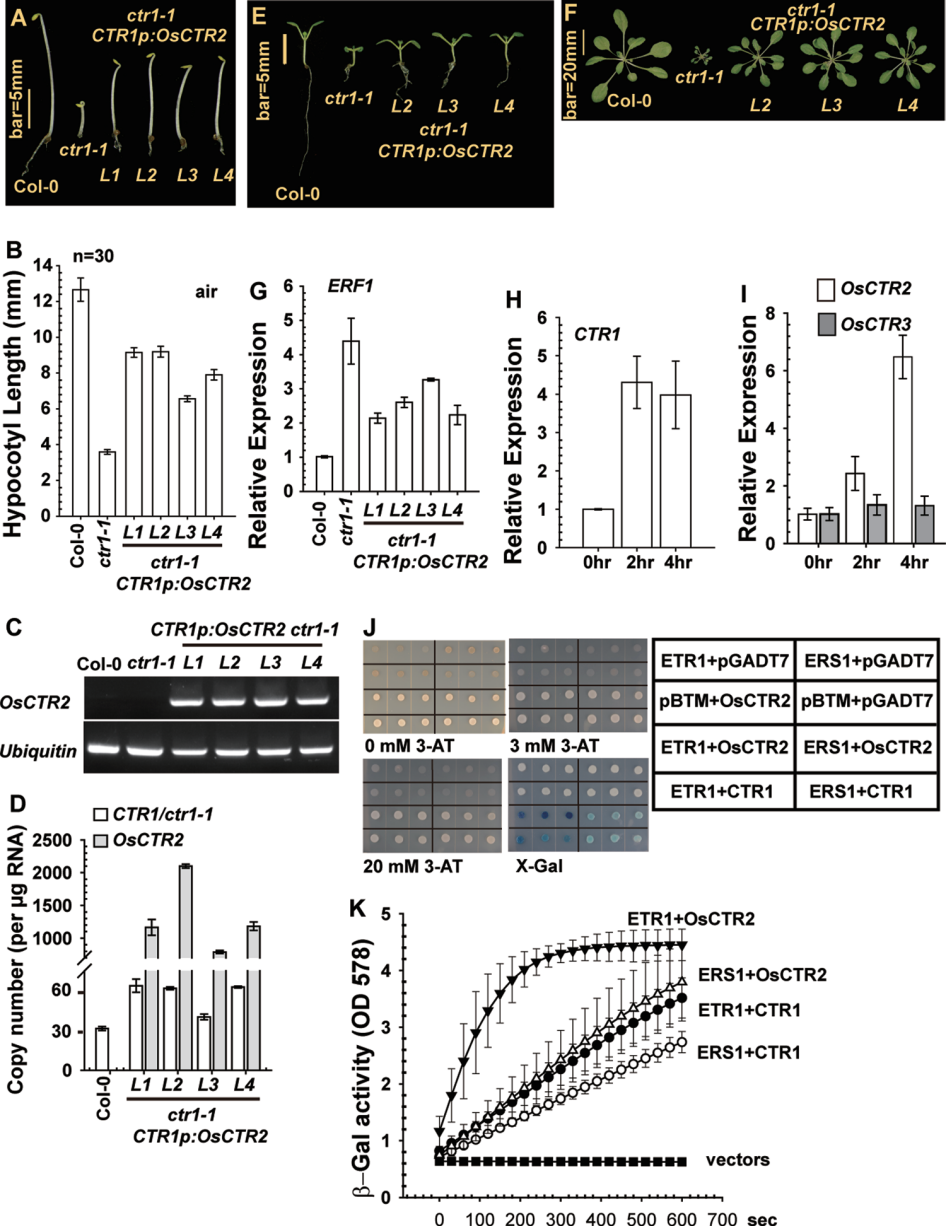
Expression of *CTR1p:OsCTR2* complements *ctr1-1*. (A, B) Phenotype (A) and hypocotyl measurements (B) of etiolated seedlings. *L*, transformation line. (C, D) Expression of the *CTR1p:OSCTR2* transgene in *ctr1-1* confirmed by RT-PCR (C) and quantified (D). (E, F) Phenotype of light-grown seedlings (E) and rosettes (F) of *ctr1-1* and *ctr1-1* expressing *CTR1p:OsCTR2*. (G) qRT-PCR analysis of mRNA level of *ERF1*. (H, I) Expression of *CTR1* in *Arabidopsis* (H) and of *OsCTR2* but not *OsCTR3* in rice (I) is ethylene inducible. (J, K) Yeast two-hybrid assay (J) and kinetics of β-galactosidase activity (K) for the interaction between ETR1/ERS1 and the OsCTR2 N terminus. Data are means ±SD of three independent experiments performed in triplicate. pBTM and pGADT7 are the vectors for the yeast two-hybrid assay.

Etiolated, air-grown *ctr1-1* seedlings showed a constitutive ethylene response phenotype, with a short hypocotyl and root and an exaggerated apical hook; wild-type (Col-0) seedlings produced a long hypocotyl and root without apical hook formation. Expression of *CTR1p:OsCTR2* rescued the *ctr1-1* seedling phenotype to a great extent, and the transgenic lines produced a long seedling hypocotyl and root without the apical hook (Fig. 2A). Measurement of seedling hypocotyls showed the same results: *ctr1-1* seedling hypocotyls were much shorter than those of *ctr1-1* seedlings expressing *CTR1p:OsCTR2* without ethylene treatment (Fig. 2B).

We confirmed expression of the *CTR1p:OsCTR2* transgene in *ctr1-1* transformation lines but not the wild type and non-transformed *ctr1-1* by RT-PCR (Fig. 2C). To gain knowledge about *OsCTR2* expression relative to the endogenous *CTR1/ctr1-1*, we estimated the copy number of *ctr1-1* and *OsCTR2* transcripts and found the *ctr1-1* and *OsCTR2* copy numbers to be higher for transgenic lines than the *CTR1* copy number in the wild type (Fig. 2D). *CTR1* expression is ethylene inducible, and the higher copy number could be explained by partial suppression of the constitutive ethylene response in the transgenic lines.

The expression of *OsCTR2* partially rescued the *ctr1-1* ethylene response phenotype. Light-grown *ctr1-1* seedlings produced small, compact cotyledons and a short root; consistently, *ctr1-1* transgenic lines expressing *CTR1p:OsCTR2* produced larger cotyledons and a longer root than *ctr1-1* seedlings (Fig. 2E). At the adult stage, expression of the *CTR1p:OsCTR2* transgene largely rescued the *ctr1-1* growth inhibition phenotype, and the transgenic lines produced a normal rosette, as in wild-type plants (Col-0) (Fig. 2F).

Expression of *ERF1* is associated with the degree of ethylene response and can be used as a marker for the ethylene response (Solano *et al.*, 1998; Liu and Wen, 2012*a*; Qiu *et al.*, 2012). By setting the *ERF1* expression level to 1 in the wild type (Col-0), the *ERF1* level in *ctr1-1* plants was higher than in *ctr1-1* lines expressing *CTR1p:OsCTR2* and in wild-type plants (Fig. 2G). Of note, expression of *CTR1* in *Arabidopsis* and *OsCTR2* but not *OsCTR3* in rice was induced by ethylene (Fig. 2H, I).

The cross-species complementation of *ctr1-1* by *CTR1p:OsCTR2* indicated that OsCTR2 could physically interact with *Arabidopsis* ethylene receptors to mediate the receptor signal output. The physical interaction of the ethylene receptors ETR1 and ERS1 with the CTR1 N terminus was shown previously by a yeast two-hybrid assay (Clark *et al.*, 1998; Huang *et al.*, 2003). As expected, the yeast two-hybrid assay revealed a physical interaction of ETR1 (residues 331–729) and ERS1 (residues 130–613) with the OsCTR2 N terminus (residues 1–513), even in the presence of high 3-amino-1,2,4-triazole concentrations as a competitive inhibitor of the *HIS3* gene product; measurement of β-galactosidase activity to quantify the interaction was consistent with X-gal staining results (Fig. 2J, K).

### Rice osctr2 mutant shows several aspects of the constitutive ethylene response phenotype

The results suggested that ectopically expressed OsCTR2 could mediate ethylene-receptor signalling in *Arabidopsis*. We expected that loss-of-function mutations of *OsCTR2* would confer constitutive ethylene responsiveness.

**Fig. 3.**
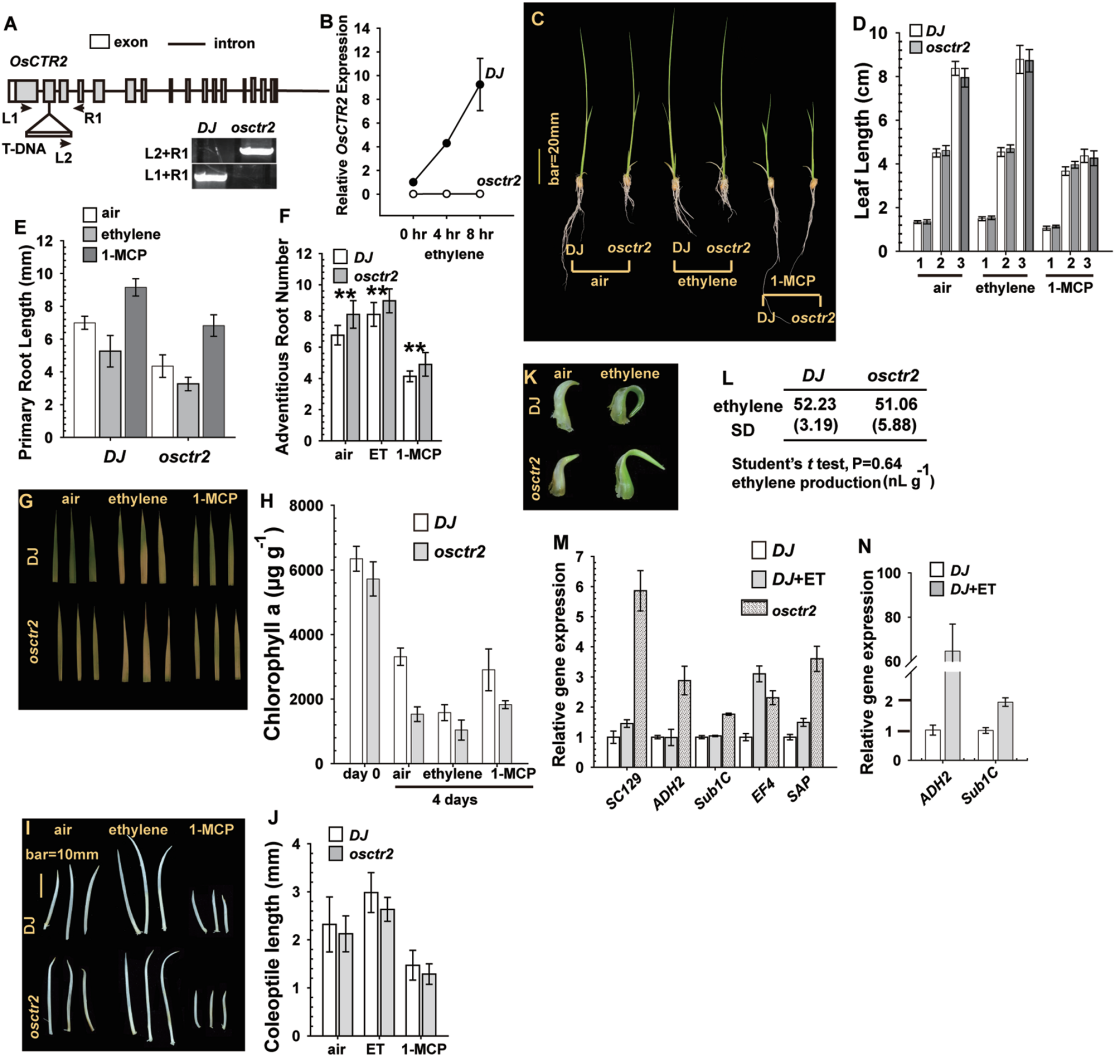
Phenotype analysis of *osctr2*. (A) Diagram of the *OsCTR2* gene structure; the T-DNA insertion site in *osctr2* is indicated. The positions of the PCR genotyping primers (L1, L2, and R1) are indicated, and the legend shows the PCR genotyping for the wild-type (DJ) and *osctr2* mutant. (B) qRT-PCR analysis of relative mRNA levels of *OsCTR2* in DJ and *osctr2* seedlings. (C, D) Seedling phenotype (C) and leaf length measurement (D) of the DJ and *osctr2* plants. Numbers on the *x*-axis in (D) indicate the order of the seedling leaves (1, first; 2, second; 3, third). (E) Primary root length in DJ and *osctr2* seedlings. (F) Number of adventitious roots in the DJ and *osctr2* seedlings. **P<0.01 for *osctr2* compared with DJ. (G, H) Leaf senescence (G) and chlorophyll *a* content (H) of DJ and *osctr2* seedlings. (I, J) Phenotype (I) and length (J) of etiolated seedling coleoptiles of DJ and *osctr2* plants. (K) Coleoptile phenotype of light-grown seedlings. (L) Ethylene evolution of DJ and *osctr2* seedlings. Data in are means ±SD of three independent experiments performed in triplicate. **P<0.01. (M, N) qRT-PCR analysis of mRNA expression of genes in rice seedlings with prolonged (M; 7 d) or short (N; 4h) ethylene treatment (100 µl l^–1^). Data are means ±SEM of three independent experiments performed in triplicate. (This figure is available in colour at *JXB* online.)

The rice *osctr2* mutation with a T-DNA insertion at the second exon of *OsCTR2* in the *japonica* variety DJ background was confirmed by PCR-based genotyping (Fig. 3A). By referencing the *OsCTR2* expression in the wild type (DJ) as 1, expression of *OsCTR2* in *osctr2* was barely detectable and was not ethylene inducible (Fig. 3B). We evaluated whether the *osctr2* allele and ethylene treatment would have the same effects on rice. Without ethylene treatment (air), the wild-type DJ and *osctr2* rice seedlings were phenotypically similar in height, with a longer root for DJ than for *osctr2* seedlings (Fig. 3C). With ethylene treatment for 7 d after germination, the root of both DJ and *osctr2* seedlings was shortened. The ethylene blocker 1-MCP substantially inhibited shoot elongation but promoted root elongation in DJ and *osctr2* seedlings.

We showed previously that ethylene treatment promoted elongation of the third leaf of the wild-type ZH11 variety (Zhang *et al.*, 2012). Measurement of individual leaves of DJ and *osctr2* showed that ethylene treatment had minor effects on leaf elongation, whereas 1-MCP treatment to block ethylene signalling reduced elongation of the third leaf to a great extent (Fig. 3D). Without ethylene treatment, DJ seedlings produced a much longer primary root than did *osctr2* seedlings. Ethylene treatment inhibited the primary root elongation in DJ and *osctr2* seedlings, with a shorter primary root in *osctr2* than DJ (Fig. 3E). 1-MCP treatment promoted primary root elongation in both DJ and *osctr2* seedlings, and *osctr2* seedlings produced a much shorter primary root than DJ seedlings (Student’s *t-*test for a paired comparison between DJ and *osctr2*, *P*<0.01).

Ethylene promotes adventitious root development in the *japonica* ZH11 variety (Zhang *et al.*, 2012). Consistently, with ethylene treatment, the adventitious root number was increased in DJ and *osctr2* seedlings, with more adventitious roots in *osctr2* than in DJ seedlings (Fig. 3F). Without ethylene, DJ seedlings produced fewer adventitious roots than *osctr2* seedlings. Treatment with the ethylene blocker 1-MCP reduced the adventitious root number to a great extent, but *osctr2* still produced more adventitious roots than DJ (Student’s *t-*test for paired comparisons between DJ and *osctr2* in each treatment, *P*<0.01).

Other aspects of the ethylene response phenotype were scored for *osctr2*. Rice leaf segments undergo senescence following ethylene treatment (Kao and Yang, 1983; Zhang *et al.*, 2012). Without ethylene treatment, *osctr2* leaves showed a much stronger senescence phenotype than DJ leaves (Fig. 3G). Ethylene treatment promoted leaf senescence in both genotypes, with a stronger senescence phenotype in *osctr2* than in DJ leaves. 1-MCP treatment delayed the senescence in DJ but not in *osctr2* plants. The degree of leaf senescence was quantified by measuring chlorophyll *a* content. Both DJ and *osctr2* leaves had a similar chlorophyll *a* content before treatment (Fig. 3H). The chlorophyll *a* content was much higher in DJ than *osctr2* leaves 4 d after detachment in air. Consistently, ethylene treatment decreased the chlorophyll *a* content in DJ and *osctr2* leaves, with more chlorophyll *a* content in DJ than in *osctr2* leaves. With 1-MCP treatment, DJ showed a much higher chlorophyll *a* content than *osctr2.*

Ethylene has been shown to promote coleoptile elongation in etiolated rice seedlings (Satler and Kende, 1985; Zhang *et al.*, 2012). The coleoptile of both DJ and *osctr2* seedlings was of similar length, and ethylene promoted but 1-MCP prevented coleoptile elongation (Fig. 3I, J). Light-grown rice seedlings produce a relatively straight coleoptile, and ethylene treatment promotes coleoptile curvature and elongation (Zhang *et al.*, 2012). Without ethylene treatment, both DJ and *osctr2* seedlings produced a relatively straight coleoptile but an exaggerated coleoptile curvature on ethylene treatment (Fig. 3K). Of note, the phenotype differences between DJ and *osctr2* were not due to differential ethylene production; both produced a similar amount of ethylene (Fig. 3L; Student’s *t-*test, *P*=0.64).

We evaluated the degree of the ethylene response in DJ and *osctr2* by measuring the expression of ethylene-inducible genes. The expression of *SUB1C*, *SC129*, and *ALCOHOL DEHYDROGENASE2* (*ADH2*) was shown previously to be induced by ethylene in the ZH11 variety (Fukao *et al.*, 2006; Zhang *et al.*, 2012). *EARLY FLOWERING4* (*EF4*; Os11g0621500) and *SENESCENCE-ASSOCIATED PROTEIN* (*SAP*; Os02g0324700) were ethylene inducible as identified by an unpublished microarray analysis. qRT-PCR revealed that, with ethylene treatment, the expression of these genes was increased to various levels in DJ seedlings and air-grown *osctr2* (Fig. 3M, N). Of note, levels of *ADH2* and *SUB1C* were not altered in DJ seedlings with prolonged ethylene treatment (7 d; Fig. 3M) but were induced with short ethylene treatment (4h; Fig. 3N).

These results suggested that, without exogenous ethylene treatment, *osctr2* seedlings showed several, but not all, aspects of the ethylene response phenotype and that these aspects were stronger than in DJ plants. Conceivably, a low basal level of ethylene responsiveness could be sufficient to maximize the elongation of seedling leaves and coleoptiles in the DJ variety, for minor effects of ethylene treatment on seedling leaf and coleoptile elongation. The expression of *ADH2* and *SUB1C* could be attenuated after prolonged treatment with a high ethylene concentration (100 µl l^–1^).

### OsCTR2^1–513^ overexpression results in various aspects of the constitutive ethylene response phenotype

The *Arabidopsis* ETR1 ethylene receptor mediates the ethylene-receptor signal output to CTR1 via interaction of the CTR1 N terminus and the ETR1 HK domain (Clark *et al.*, 1998). Excess CTR1 N terminus (residues 7–560) prevents receptor signalling, and the *CTR1* ^*7–560*^ overexpressor *CTR1Nox* produces a constitutive ethylene response phenotype (Huang *et al.*, 2003; Qiu *et al.*, 2012). Involvement of OsCTR2 in the ethylene-receptor signalling could be evaluated by determining whether overexpression of the OsCTR2 N terminus also results in a constitutive ethylene response.

**Fig. 4.**
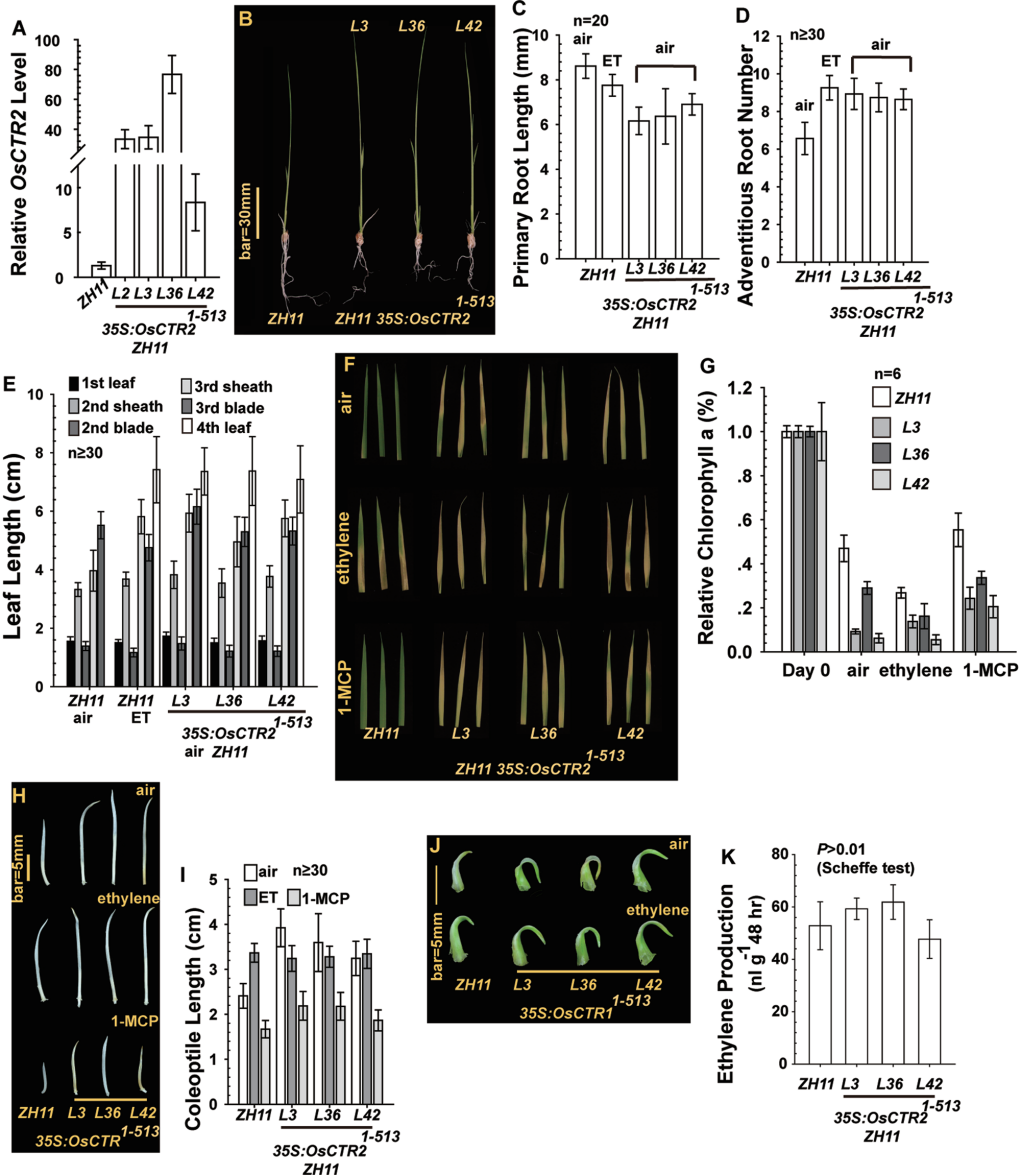
Expression of *35S:OsCTR2* ^*1–513*^ confers a constitutive ethylene response in rice. (A–E) qRT-PCR analysis of *OsCTR2* mRNA level (A), seedling phenotype (B), primary root length (C), adventitious root number (D), and seedling leaf length (E) of the wild type (ZH11) and *35S:OsCTR2* ^*1–513*^ transgenic lines. (F–I) Leaf senescence phenotype (F), relative chlorophyll *a* content (G), coleoptile phenotype (H), and coleoptile length (I) of the wild type (ZH11) and transgenic lines. (J) Coleoptile phenotype of light-grown seedlings. (K) Ethylene evolution in the wild-type (ZH11) and transgenic lines. Data are means ±SD of three independent experiments performed in triplicate. L, transformation line. (This figure is available in colour at *JXB* online.)

*35S:OsCTR2* ^*1–513*^, which encodes the OsCTR2 N terminus, was transformed into the *japonica* rice variety ZH11. Expression of the *35S:OsCTR2* ^*1–513*^ transgene in four representative transgenic lines was confirmed by qRT-PCR, and lines L3, L36, and L42 were selected for further study because of their differential *OsCTR2* ^*1–513*^ levels (Fig. 4A). The wild type (ZH11) showed a shorter seedling and longer roots than the transformation lines (Fig. 4B). Measurement of primary roots gave the same results: the primary root was longer for ZH11 than for the *35S:OsCTR2* ^*1–513*^ transgenic lines (Scheffe’s test, *P*<10^–8^). The primary root was shorter in ZH11 with than without ethylene treatment (Scheffe’s test, *P*=0.0141; Fig. 4C). We showed previously that ethylene treatment increases the number of adventitious roots. As expected, without ethylene treatment, ZH11 seedlings produced fewer adventitious roots than the *35S:OsCTR2* ^*1–513*^ transgenic lines and ethylene-treated ZH11 seedlings (Scheffe’s test, *P*<10^–18^). The number of adventitious roots was similar for ethylene-treated ZH11 and *35S:OsCTR2* ^*1–513*^ transgenic lines (Scheffe’s test, *P*=0.04–0.588; Fig. 4D).

Ethylene treatment promoted sheath elongation of the third leaf in ZH11 seedlings (Zhang *et al.*, 2012). As expected, third-leaf sheaths of ethylene-treated ZH11 seedlings and air-grown *35S:OsCTR2* ^1–513^ lines were 1–2cm longer than those of non-treated ZH11 seedlings (Scheffe’s test, *P*<10^–6^; Fig. 4E). Consistent with our previous finding that ethylene promoted the growth of the fourth leaf (Zhang *et al.*, 2012), ethylene-treated ZH11 and air-grown transgenic lines but not air-grown ZH11 produced four leaves (Fig. 4E).

These results suggested that overexpression of the OsCTR2 N terminus results in a constitutive ethylene response. Conceivably, leaf senescence in the transgenic lines could occur without ethylene treatment. ZH11 seedling leaf segments showed a mild senescence phenotype 4 d after detachment in air, and 1-MCP treatment prevented the senescence phenotype (Fig. 4F). Ethylene treatment for 4 d promoted the senescence progression, and ZH11 leaf segments showed a strong senescence phenotype. For the *35S:OsCTR2* ^*1–513*^ transgenic rice lines, the leaf segments showed a strong senescence phenotype 4 d after detachment in air, and 1-MCP did not prevent the senescence phenotype. With ethylene treatment for 4 d, the transgenic lines produced the same senescence phenotype as with no treatment (Fig. 4F). To quantify the degree of leaf senescence, we measured relative chlorophyll *a* content after the senescence test. By setting the chlorophyll *a* content to 1 in leaves before treatment, leaf segments of the *35S:OsCTR2* ^*1–513*^ transgenic rice lines showed much lower relative chlorophyll *a* content than ZH11 plants in all treatments (Fig. 4G). 1-MCP treatment attenuated the chlorophyll *a* reduction in ZH11 leaf segments, and the effect was minor in leaves of transgenic lines.

Ethylene promotes the coleoptile elongation of etiolated rice seedlings (Satler and Kende, 1985; Zhang *et al.*, 2012). As expected, etiolated ZH11 seedlings produced a longer coleoptile with than without ethylene treatment, and etiolated seedlings of the *35S:OsCTR2* ^*1–513*^ transgenic lines produced a longer coleoptile than ZH11 seedlings; 1-MCP treatment reduced the coleoptile length, and ZH11 produced a much shorter coleoptile than the transgenic lines (Fig. 4H, I). Grown under light, ZH11 seedlings produced a relatively straight coleoptile compared with seedlings with ethylene treatment; consistently, *35S:OsCTR2* ^*1–513*^ transgenic lines produced a coleoptile with curvature, regardless of ethylene treatment (Fig. 4J). ZH11 and the *35S:OsCTR2* ^*1–513*^ transgenic lines produced the same amount of ethylene (Scheffe’s test, *P*>0.01; Fig. 4K). Thus, the *35S: OsCTR2* ^*1–513*^ transgene facilitated a constitutive ethylene response without increasing ethylene production.

### Green fluorescent protein (GFP)-fused OsCTR2 co-localizes with the ER marker ER-rk

Previous studies of the subcellular localization of *Arabidopsis* CTR1 and tomato CTR1-related proteins revealed their localization at the ER (Gao *et al.*, 2003; Zhong *et al.*, 2008). We studied the subcellular localization of OsCTR2 with GFP-fused OsCTR2.

**Fig. 5.**
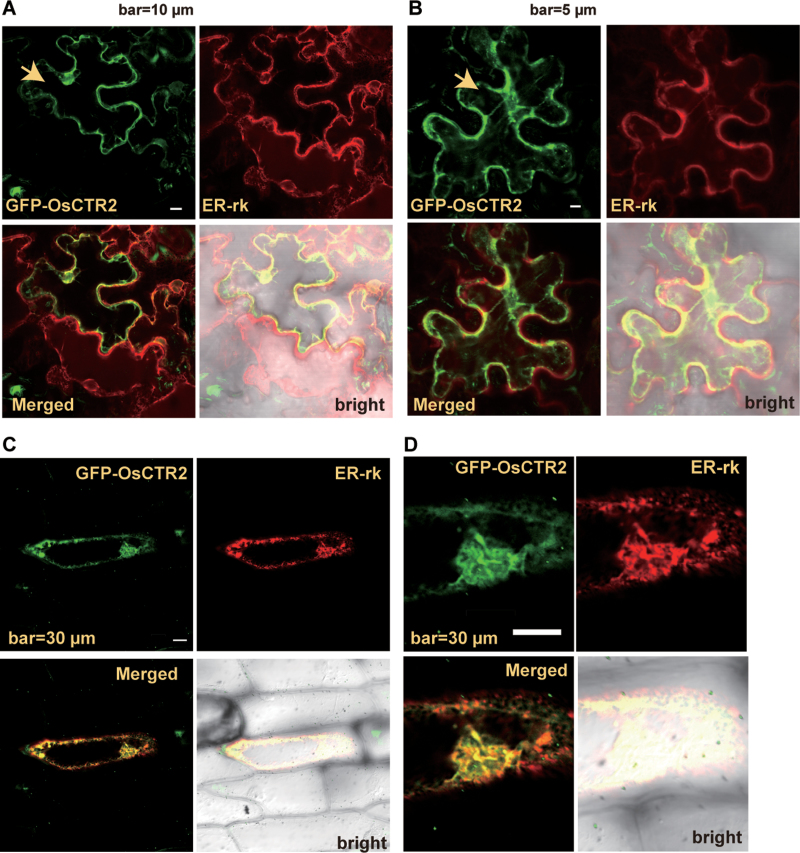
Subcellular localization of GFP–OsCTR2. Fluorescence of GFP–OsCTR2 (green) and ER-rk (red) in tobacco (A, B) and onion epidermal (C, D) cells. The cell for the co-localization study is indicated with an arrowhead in (A) and (B).

The transgene encoding the full-length GFP-fused OsCTR2 was transformed into *Arabidopsis* expressing the ER marker ER-rk (Nelson *et al.*, 2007). Unfortunately, the resulting transformants did not show fluorescence by ER-rk, possibly because of co-suppression. We determined whether the two fluorescent proteins could be co-expressed in tobacco cells by infiltration with a mix of *Agrobacterium* containing both clones. The two proteins did not express equally, and we selected only cells expressing both proteins at a similar level. Green (GFP–OsCTR2) and red (ER-rk) fluorescence was observed mainly surrounding the nucleus, at the cell edge of tobacco cells, with an uneven, discrete pattern (Fig. 5A), and the ER network was also observed (Fig. 5B). To obtain other evidence supporting localization of OsCTR2 at the ER, GFP–OsCTR2 was transiently co-expressed with ER-rk in onion epidermal cells by particle bombardment. Consistently, the fluorescence of GFP–OsCTR2 and ER-rk co-localized (Fig. 5C). The nucleus was surrounded by GFP–OsCTR2 and ER-rk (Fig. 5D), which is consistent with the close association of the ER with the outer membrane of the nuclear envelope.

### Evaluation of ethylene effects on agronomic traits involving yield

The growth conditions for rice are highly demanding, and evaluating ethylene effects on agronomic traits with prolonged ethylene treatment without compromising rice growth is technically difficult. With the higher degree of ethylene responsiveness in *osctr2* and *35S:OsCTR2* ^*1–513*^ lines than in the wild type, we evaluated the effects of the constitutive ethylene response, which could mimic a prolonged ethylene treatment, on yield-related agronomic traits.

**Fig. 6.**
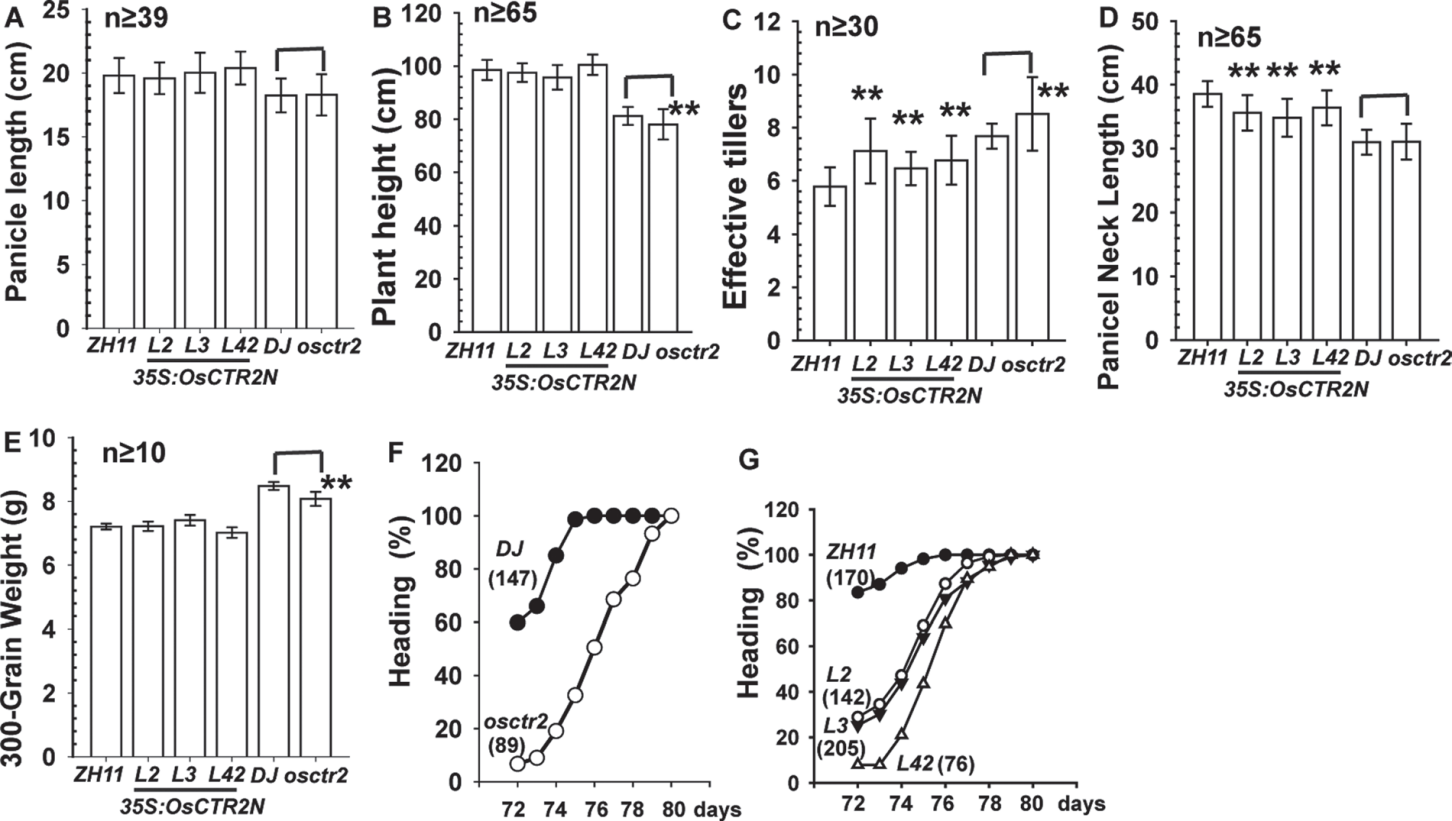
Quantification of yield-related agronomic traits. (A) Panicle length, (B) plant height, (C) effective tiller number, (D) panicle neck length, (E) 300-grain weight (gram), and (F) and (G) heading status of wild-type varieties (DJ and ZH11), *osctr2*, and *35S:OsCTR2* ^*1–513*^ transgenic lines. Data are means ±SD of three independent experiments performed in triplicate. **P<0.01. Sample sizes are as indicated or are shown in parentheses.

Both DJ and *osctr2* seedlings produced a panicle of the same length (Fig. 6A; Student’s *t*-test, *P*=0.88); consistently, ZH11 and the *35S:OsCTR2* ^*1–513*^ transgenic lines produced a panicle of the same length (Fig. 6A; Scheffe’s test, *P*>0.039). DJ plants were slightly taller than *osctr2* plants (Fig. 6B; Student’s *t*-test, *P*<10^–5^); differences in plant height between ZH11 and the *35S:OsCTR2* ^*1–513*^ transgenic lines were minor, and the ZH11, L2, and L42 lines were the same height (Fig. 6B; Scheffe’s test, *P*>0.026). Effective tillers produce panicles, and the number of effective tillers is an important trait determining rice yield. DJ plants produced fewer effective tillers than *osctr2* plants (Fig. 6C; Student’s *t-*test, *P*=0.002); consistently, ZH11 produced fewer effective tillers than the *35S:OsCTR2* ^*1–513*^ transgenic lines (Fig. 6C; Scheffe’s test, *P*<0.01). The constitutive ethylene response could increase the number of effective tillers. The panicle neck length (or the upper-most internode length) is an important trait determining rice plant height. The panicle neck length of DJ and *osctr2* plants was identical (Fig. 6D; Student’s *t*-test, *P*=0.89), whereas that for ZH11 was 2–3.7cm longer than for the transgenic lines (Fig. 6D; Scheffe’s test, *P*<10^–4^). Grain weight is also an important trait that determines yield. We measured the weight of 300 grains; those of DJ plants had a slightly greater weight than those of *osctr2* plants (Fig. 6E; Student’s *t*-test, *P*<10^–4^) and the 300-grain weight of ZH11 and the *35S:OsCTR2* ^*1–513*^ transgenic lines was identical (Fig. 6E; Scheffe’s test, *P*>0.04).

Overexpression of the ethylene receptor-like gene *OsETR2* results in delayed flowering in ZH11 and reduced sensitivity to ethylene, and alterations in the ethylene response may result in the delayed flowering phenotype (Wuriyanghan *et al.*, 2009). We determined whether *osctr2* and *35S:OsCTR2* ^*1–513*^ expression affected heading time. Normally, the heading of field-grown rice plants is not synchronous; we scored the heading status within 72–80 d post-planting. At day 72, DJ plants (60%) showed early heading compared with *osctr2* plants (6.7%). At day 74, heading was complete in DJ plants but not until day 80 in *osctr2* plants (Fig. 6F). At day 72, ZH11 (85.5%) also showed early heading compared with the *35S:OsCTR2* ^*1–513*^ transgenic lines (7.9–28.9%). At days 75 and 76, heading was complete for ZH11 but not until day 79 for the *35S:OsCTR2* ^*1–513*^ transgenic lines (Fig. 6G).

We showed an association of an increase in effective tiller number and delayed heading with *osctr2* (in DJ) and *35S:OsCTR2* ^*1–513*^ expression (in ZH11). Elevation in the degree of the constitutive ethylene response may be associated with these traits.

## Discussion

*Arabidopsis* CTR1 is a key component mediating the ethylene-receptor signal output to suppress ethylene responses (Ju *et al.*, 2012; Qiao *et al.*, 2012). Our phylogenetic analysis suggested that rice has two *CTR1*-related genes: *OsCTR2* and the annotated *OsCTR1*. Without mRNA evidence for *OsCTR1*, the presence of the *OsCTR1* locus and the OsCTR1 protein sequence remain to be determined. Our data support the suggestion that OsCTR2 can mediate ethylene-receptor signalling in rice.

Several lines of evidence suggest that OsCTR2 is not the only component that can mediate the ethylene-receptor signal output. The mutant *osctr2* showed a similar leaf growth phenotype to the wild type (DJ), with or without treatment with ethylene and its blocker 1-MCP. The ethylene-promoted coleoptile growth and curvature phenotype was also similar between DJ and *osctr2* plants, and 1-MCP treatment inhibited coleoptile elongation in both genotypes. Therefore, the ethylene-induced leaf and coleoptile growth phenotype could be independent of OsCTR2. However, ethylene treatment and the *osctr2* allele had the same effects on primary root growth, adventitious root development, and leaf senescence, so these ethylene-induced growth alterations involved OsCTR2. The involvement of OsCTR2 in ethylene signalling appeared to be tissue specific.

Although leaf and coleoptile growth was not affected by *osctr2* at the seedling stage, *35S:OsCTR2* ^*1–513*^ expression had the same effects as ethylene treatment on these phenotypes and other aspects of ethylene-induced alteration in growth. An excess amount of the CTR1 N terminus prevents ethylene-receptor signalling, and thus the ethylene response is relieved from suppression (Huang *et al.*, 2003; Qiu *et al.*, 2012). We hypothesized that *35S:OsCTR2* ^*1–513*^ expression could result in a gain of function to prevent ethylene-receptor signalling in ZH11, such that various aspects of the ethylene response phenotype were observed in *35S:OsCTR2* ^*1–513*^ lines.

Our results suggested that OsCTR2 is not the only component that mediates the ethylene-receptor signal output in rice. The putative OsCTR1 is highly related to CTR1 and could have a role in ethylene-receptor signalling. *OsCTR1* and *OsCTR2* could be expressed differentially in various tissues because *osctr2* showed several but not all aspects of the ethylene-induced growth alteration in a tissue-specific manner. Alternatively, OsCTR1 and OsCTR2 could be functionally divergent to suppress various aspects of the ethylene response. The latter scenario suggests the presence of distinct modules that could mediate ethylene-receptor signalling. Interestingly, the use of modules with distinct and overlapping functions in ethylene signalling has also been found in tomato. *Arabidopsis* RTE1 promotes the ethylene-receptor ETR1 signal output. In tomato, the ethylene response is differentially controlled by two RTE1-related proteins: GREEN-RIPE and GREEN-RIPE LIKE1 (Ma *et al.*, 2012). Moreover, in *Arabidopsis*, ethylene-receptor signalling can be mediated by a pathway independent of CTR1 (Qiu *et al.*, 2012; Xie *et al.*, 2012). Ethylene-receptor signalling in rice could be also mediated by a pathway independent of CTR1-related proteins. The mediation of ethylene-receptor signalling by distinct modules or to alternative signalling pathways implies a multilevel regulation of ethylene signalling.

CTR1 does not have any predicted transmembrane helices and is co-fractionated with the ER membrane (Gao *et al.*, 2003). A subcellular localization study involving yellow fluorescent protein (YFP)-tagged tomato CTR1-related proteins suggests that each N terminus (LeCTR-N) of the three tomato CTR1-related proteins LeCTR1, LeCTR3, and LeCTR4 interacts with the ethylene receptor NEVER-RIPE (NR) in onion epidermal cells. Without NR co-expression, the YFP–LeCTR-N fusions were localized to the cytoplasm and nucleus but not to the ER (Zhong *et al.*, 2008). The subcellular localization of a fluorescent protein-tagged full-length CTR1-related protein remains to be determined. Although we were unable to directly show the subcellular localization of GFP–OsCTR2 in rice cells, we showed a co-localization of GFP–OsCTR2 in onion and tobacco cells with the ER marker ER-rk. Moreover, complementation of *Arabidopsis ctr1-1* by *CTR1p:OsCTR2* and the physical interaction between ETR1/ERS1 and the OsCTR2 N terminus support the suggestion that OsCTR2 is localized at the ER by associating with ethylene receptors. In contrast to the localization of YFP–LeCTR-Ns to the cytoplasm and nucleus in onion cells, GFP–OsCTR2 was not observed in the nucleus. The localization of YFP–LeCTR-Ns to the nucleus could be an artefact; alternatively, LeCTRs could shuttle between the ER and the nucleus, but the functional significance of this needs to be addressed.

A few studies of ethylene effects on growth in *japonica* varieties were conducted mainly with the ethylene precursor ACC and the ethylene releaser ethephon to replace ethylene treatment (Jun *et al.*, 2004; Mao *et al.*, 2006; Wuriyanghan *et al.*, 2009). ACC is rapidly consumed after its application, and ethephon hydrolyses into ethylene and strong acids. Of note, ethylene production cannot be controlled experimentally with ACC and ethephon, and for ethylene effects that require a prolonged response window, ACC and ethephon are not ideal as replacements for ethylene (Lavee and Martin, 1981; Zhang and Wen, 2010; Zhang *et al.*, 2010). Silver is an effective blocker of the ethylene response, and silver treatment results in abnormal root growth in rice seedlings, with accumulation of a dark-brown unknown compound. Whether silver can be an ideal ethylene blocker without affecting normal rice growth needs to be evaluated.

Evaluating the effects of ethylene on agronomic traits of rice plants is technically difficult. With the constitutive ethylene response, the effect of prolonged ethylene treatment on agronomic traits related to yield could be evaluated by comparing phenotypic differences between the wild-type (DJ) and *osctr2* plants and between ZH11 and the *35S:OsCTR2* ^*1–513*^ lines. We found two agronomic traits related to yield that were altered with ethylene: both *osctr2* and the *35S:OsCTR2* ^*1–513*^ lines produced more effective tillers and showed delayed flowering compared with the wild type (DJ and ZH11). With more effective tillers, rice yield could be increased.

The effect of ethylene on rice growth and development inferred from *osctr2* and the *35S:OsCTR2* ^*1–513*^ transgenic lines was consistent with the effect of treatment with ethylene and 1-MCP and that inferred from studies of ethylene-insensitive *35S:OsRTH1* lines (Zhang *et al.*, 2012). The isolation of *osctr2*, together with the *35S:OsCTR2* ^*1–513*^ lines and *35S:OsRTH1* lines that we obtained previously should facilitate future studies of the underlying mechanisms of the ethylene effect on various aspects of rice growth and development in response to external and internal cues.

## Supplementary data

Supplementary data are available at *JXB* online

Supplementary data S1. Primer sets for cloning and qRT-PCR.

Supplementary Data

## Abbreviations:

1-MCP: 1-methylcyclopropene
ACC: 1-aminocyclopropane-1-carboxylic acid
ER: endoplasmic reticulum
GFP: green fluorescent protein
qRT-PCR: quantitative reverse transcription-PCR
X-gal: 5-bromo-4-chloro-indolyl-β-d-galactopyranoside
YFP: yellow fluorescent protein.
