# Supplementary material for: Rice CONSTITUTIVE TRIPLE-RESPONSE2 is involved in the ethylene-receptor signalling and regulation of various aspects of rice growth and development
Source: J Exp Bot. 2013 Sep 4;64(16):4863–75. doi: 10.1093/jxb/ert272 (PMC3830475; doi:10.1093/jxb/ert272)
Supplement: Supplementary Data [file supp_64_16_4863__index.html]

Rice CONSTITUTIVE TRIPLE-RESPONSE2 is involved in the ethylene-receptor signalling and regulation of various aspects of rice growth and development — Rice CONSTITUTIVE TRIPLE-RESPONSE2 is involved in the ethylene-receptor signalling and regulation of various aspects of rice growth and development — Supplementary Data 

# Rice CONSTITUTIVE TRIPLE-RESPONSE2 is involved in the ethylene-receptor signalling and regulation of various aspects of rice growth and development

## Supplementary Data

Data files

**Files in this Data Supplement:**

- Supplementary Data - Supplementary Data
