# Supplementary material for: Rice CONSTITUTIVE TRIPLE-RESPONSE2 is involved in the ethylene-receptor signalling and regulation of various aspects of rice growth and development
Source: J Exp Bot. 2013 Sep 4;64(16):4863–75. doi: 10.1093/jxb/ert272 (PMC3830475; doi:10.1093/jxb/ert272)
Supplement: Supplementary Data [file supp_ert272_jexbot099457_file001.pdf]

Supplemental data S1. Primer sets for cloning and qRT-PCR.

**Primers**	**sequences**
AtCTR1OF	5'-atgtcgacGTTCTCGGCATGAATCAT-3'
AtCTR1PR	5'-atgaattcGGAAAAGAGAGTTATCGCG-3'
OsCTR2-F	5'- ATAGATCTATGAAGGCCGACGCCAAG-3'
OsCTR2-R	5'-ATAGATCTTCAAGGGCTCGTATCTTC-3'
OsCTR2-N-F	5'-ATGAAGGCCGACGCCAAG-3'
OsCTR2-NR	5'-CAGGATTACAAACCTGTC-3'
OsCTR2-F	5'-GAAGATTAGTTCAGGTTCTG-3'
OsCTR2-R	5'-CAATCAGATATTCCCTGTCA-3'
OsCTR3-F	5'-GGTTTGTAAGGGAGTACGTCGT-3'
OsCTR3-R	5'-CTTAGCAACTGAATACTGAGGA-3'
EF4-F	5'-AGCTTCTCAGGGATGGCCAAT-3'
EF4-R	5'- CGACGTTCCGGGTGAGGTTGT-3'
SAP-F	5'- GCGGATCCGACGCGCTCA-3'
SAP-R	5'- GCTGAGGAGGAACTTGTTCA-3'
Sub1C-F	5'- CTGCTCCGACGACCTGAT-3'
Sub1C-R	5'- TTAGGCGAGTCGCATGTCAA-3'
ADH2-F	5'- CCCATCCCTGGATTCAGGT-3'
ADH2-R	5'- CACGAGGTAGGTGCTGATTGA-3'
SC129-F	5'- TGACGGTGTACGGTCCGAT-3'
SC129-R	5'- TCGGCGTACTGGTCACAGAT-3'
OsAct-F	5'-GAAGATCACTGCCTTGCTCC-3'
OsAct-R	5'- CGATAACAGCTCCTCTTGGC-3'
UBI-F	5'-ATGGAAAATCCCACCTACTAAATT-3'
UBI-R	5'-TTG AACAACTCGTAGCAACTCATC-3'
CTR1-F	5'-CATCGTTTCTGGGTTAATGGCT-3'
CTR1-R	5'-TCCTTGAAGGCTGGATCACTAC-3'

For *OsCTR2* expression measurement, the primer set was OsCTR2-F and OsCTR2-R. Expression of *OsCTR3* was measured with the primer set OsCTR3-F and OsCTR3-R. EF4-F and EF4-R were for *EF4* (Os 11g0621500) expression measurement. *SAP* (Os02g0324700) expression was measured with the primer set SAP-F and SAP-R. *Sub1C* expression was measured with the primer set Sub1C-F and Sub1C-R. The primer set ADH2-F and ADH2-R was for *ADH2* expression measurement. *SC129* expression was measured by SC129-F and SC129-R. The internal calibrator for rice gene expression was OsActin with the primer set was OsAct-F and OsAct-R. For Arabidopsis *ERF1* measurement, *Ubiquitin* was used as the internal calibrator, with

the primer set UBI-F and UBI-R. Arabidopsis CTR1 copy number was measured with the primer set CTR1-F and CTR1-R.
